# P-1687. Evaluation and Characterization of a Novel DNA Lysis Technique for Extraction of Nontuberculous Mycobacteria DNA

**DOI:** 10.1093/ofid/ofaf695.1861

**Published:** 2026-01-11

**Authors:** Monique Gainey, Alexia Stettinius, Hal Holmes, Emmanuel Naatei Nartey, Sydney Stayrook, Adam Maxwell, Eli Vlaisavljevich, Jayasimha Rao

**Affiliations:** Virginia Tech Carilion School of Medicine, Roanoke, VA; Virginia Tech, Blacksburg, Virginia; Virginia Polytechnic Institute and State University, Blacksburg, Virginia; Virginia Tech Carilion School of Medicine, Roanoke, VA; Virginia Tech Carilion School of Medicine, Roanoke, VA; Virginia Polytechnic Institute and State University, Blacksburg, Virginia; Virginia Polytechnic Institute and State University, Blacksburg, Virginia; Carilion Clinic/Virginia Tech Carilion School of Medicine, Roanoke, Virginia

## Abstract

**Background:**

From enzymatic buffers to boiling methods, the complexity of *Mycobacterium’s* cell wall has challenged traditional lysis techniques in extracting adequate DNA for downstream analyses. A novel focused ultrasound extraction (FUSE) system was developed to address such gaps. FUSE uses a miniaturized benchtop focused ultrasound transducer to create cavitation clouds that mechanically lyse targeted samples (Fig 1). Previous studies have proven that this innovative system can successfully lyse cell walls of complex plant tissue. The aim of this study is to determine whether FUSE can rapidly lyse *Mycobacterium’s* cell wall and release DNA at greater yields compared to standard extraction methods.

**Figure:**
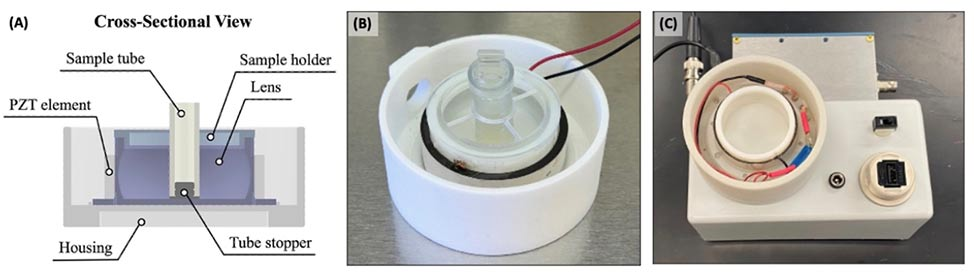
FUSE Setup. FUSE uses a cylindrical, piezoelectric (PZT) transducer to generate cavitation clouds that mechanically lyse samples (A; B). The transducer is placed on top of driving electronics (C) which powers the system.

**Figure:**
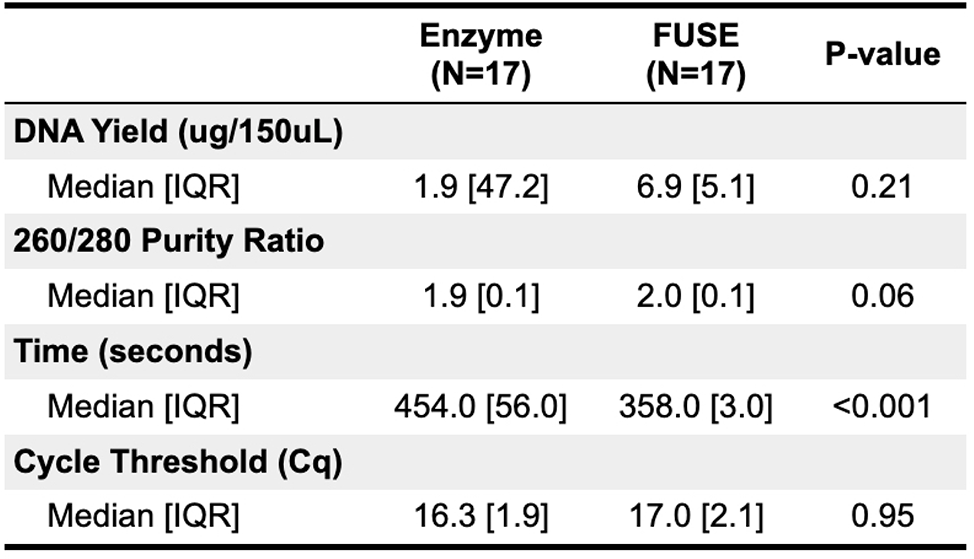
Comparison of primary outcomes between each treatment group. 260/280 purity ratio of 1.8 is accepted as pure DNA. Cq values below 30 were considered to indicate high abundance of target nucleic acid.

**Methods:**

Seventeen cultured pellets of *Mycobacterium smegmatis* (*M. smegmatis*) were treated with a 750kHz FUSE transducer at 200 pulses per second with a pulse dose of 2500 in a Qiagen B1 buffer, RNAse, and proteinase K suspension, while pellets in the comparison group were incubated in the same suspension plus lysozyme for 30 minutes. DNA was isolated using gravity flow columns, precipitated with 100% isopropanol, and purified with 70% ethanol. DNA yield (μg/μl) and 260/280 purity ratio were measured using NanoDrop spectrophotometer and were amplified by qPCR using SYBR green *M. smegmatis* 16S ribosomal DNA primer sets.

**Figure:**
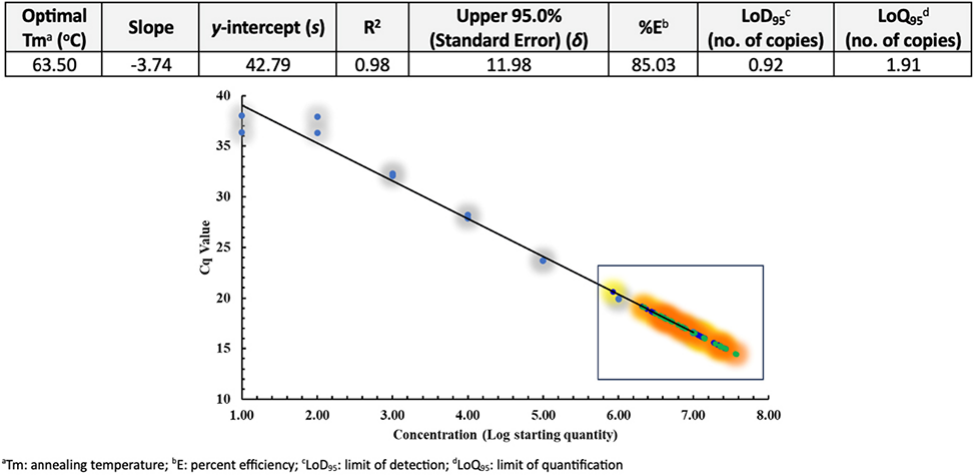
Optimization Performance and qPCR Characterization. The standards of each target were compared with standard curve reactions performed in duplicates from two plates. Standard curve shows copies/20μl reaction and is expressed in log starting quantity (gray). Extracts were diluted by a factor of 10. The box highlights the plot of each Cq value for both treatment groups with the lysozyme group represented in yellow and FUSE in orange. A Cq of 37.11 and 1.52 are considered as cutoff line for the detection limit.

**Results:**

With a median DNA yield of 6.9 μg/150 μl (IQR 5.1), samples treated with FUSE released more DNA across 17 samples compared to the lysozyme group (1.9 μg/150 μl (IQR 47.2)). Both lysing methods had acceptable purity ratio, suggesting low protein contaminants (Table 1). Lysis time under FUSE was 12.5 seconds, which shortened the overall median extraction time by 96 minutes (p-value < 0.001; Fig 2). Low cycle threshold values for the lysozyme and FUSE groups indicate high amounts of *M. smegmatis* DNA and target gene amplification with no significant difference between the groups (Fig 2).

**Conclusion:**

FUSE consistently released more DNA at a faster rate and with less variability in yield, demonstrating the feasibility of integrating extraction protocols with FUSE to effectively and efficiently lyse *M. smegmatis*’ samples. Future directions aim to determine whether FUSE can release *Mycobacterium tuberculosis* DNA from sputum samples and develop point of care FUSE diagnostic systems.

**Disclosures:**

All Authors: No reported disclosures

